# Involvement of the LcARF17- and LcRAP2-4-LcLOX7 regulatory modules in the biosynthesis of fresh aroma in litchi aril

**DOI:** 10.1093/hr/uhag010

**Published:** 2026-01-09

**Authors:** Zhuoyi Liu, Yimeng Wang, Hang Zhang, Zidi He, Zhiqi Li, Ke Ma, Minglei Zhao, Jianguo Li, Xingshuai Ma

**Affiliations:** Key Laboratory of Biology and Genetic Improvement of Horticultural Crops (South China), Ministry of Agriculture and Rural Affairs, Guangdong Litchi Engineering Research Center, College of Horticulture, South China Agricultural University, Guangzhou 510642, China; Key Laboratory of Biology and Genetic Improvement of Horticultural Crops (South China), Ministry of Agriculture and Rural Affairs, Guangdong Litchi Engineering Research Center, College of Horticulture, South China Agricultural University, Guangzhou 510642, China; Key Laboratory of Biology and Genetic Improvement of Horticultural Crops (South China), Ministry of Agriculture and Rural Affairs, Guangdong Litchi Engineering Research Center, College of Horticulture, South China Agricultural University, Guangzhou 510642, China; Institute of Fruit Tree Research, Guangdong Academy of Agricultural Sciences, Guangzhou 510640, China; Beijing Key Laboratory of Development and Quality Control of Ornamental Crops, Department of Ornamental Horticulture, China Agricultural University, Beijing 100193, China; Sanya Institute, Department of Horticulture, China Agricultural University, Sanya 572024, China; Dongguan Agricultural Scientific Research Center, Department of Agriculture and Rural Affairs of Dongguan, Dongguan 523086, China; Key Laboratory of Biology and Genetic Improvement of Horticultural Crops (South China), Ministry of Agriculture and Rural Affairs, Guangdong Litchi Engineering Research Center, College of Horticulture, South China Agricultural University, Guangzhou 510642, China; Key Laboratory of Biology and Genetic Improvement of Horticultural Crops (South China), Ministry of Agriculture and Rural Affairs, Guangdong Litchi Engineering Research Center, College of Horticulture, South China Agricultural University, Guangzhou 510642, China; Key Laboratory of Biology and Genetic Improvement of Horticultural Crops (South China), Ministry of Agriculture and Rural Affairs, Guangdong Litchi Engineering Research Center, College of Horticulture, South China Agricultural University, Guangzhou 510642, China

## Abstract

Fatty acid-derived volatile organic compounds (VOCs), especially C6 and C9 aldehydes and alcohols, are vital contributors to the fresh aroma of fruits. However, the specific volatiles responsible for this freshness and their biosynthetic regulatory mechanisms remain poorly characterized in litchi (*Litchi chinensis* Sonn.). In this study, we systematically characterized the VOC profiles of 24 representative litchi cultivars and identified four critical compounds—*trans,cis*-2,6-nonadien-1-ol, 1-hexanol, (*E*)-6-nonenal, and (*E*)-2-hexen-1-ol—as primary determinants of fresh-aroma variation. Weighted gene co-expression network analysis of the transcriptomic data, corroborated by RT-qPCR, revealed a strong positive correlation between the expression of *LcLOX7* and the abundance of these key fresh-aroma volatiles. Functional characterization via *LcLOX7* overexpression in litchi callus and tomato fruits validated its pivotal role in enhancing the biosynthesis of fatty acid-derived VOCs, specifically C9 volatiles. Subsequent *in vivo* and *in vitro* assays confirmed the direct transcriptional activation of *LcLOX7* by two transcription factors (TF), *LcARF17* and *LcRAP2-4*. The expression patterns of these TFs correlated with the accumulation of key fresh-aroma volatiles across different litchi cultivars and paralleled *LcLOX7* expression during fruit ripening. Moreover, overexpression and silencing of *LcARF17* or *LcRAP2-4* in litchi callus validated their regulatory function in promoting C9 volatile synthesis. Our findings collectively support a regulatory model wherein the LcARF17/LcRAP2-4–LcLOX7 module orchestrates the biosynthesis of fresh aroma in litchi fruit. Notably, this study provides the first evidence that ARF transcription factor participates in the formation of fresh fruit aroma, thereby offering novel insights for the molecular breeding of flavor quality in fruit crops.

## Introduction

Litchi (*Litchi chinensis* Sonn.), a member of the Sapindaceae family, is an economically and culturally significant tropical and subtropical fruit tree native to Yunnan Province, China [1]. Litchi is valued worldwide for its bright scarlet pericarp, translucent and succulent aril, delicate texture, and abundant nutritional components. However, it is further distinguished by its unique aromatic qualities, positioning it as a high-value horticultural crop [2]. Aroma quality serves as a key determinant of consumer preference and market competitiveness in litchi. Prior studies have identified terpenoids and alcohols as the major contributors to its characteristic rose-like and citrus-like aroma [3]. After millennia of natural evolution and selective breeding, litchi has diversified into hundreds of cultivars with distinct aroma profiles—for instance, ‘Bingli’ exhibits a honey-like fragrance [4], ‘Guiwei’ emits osmanthus-like aromas [5], and ‘Guanyinlv’ is characterized by a fresh and clean scent [6]. Despite this diversity and significance, the molecular mechanisms underlying aroma biosynthesis in litchi, particularly those regulating fresh-scent volatiles, remain largely uncharacterized.

Fruit aroma results from species-specific compositions of low-molecular-weight volatile organic compounds (VOCs) [7], which are biosynthesized through four core metabolic pathways: terpene, phenylpropanoid, fatty acid, and branched-chain amino acid metabolism [8–11]. Among these, fatty acid-derived VOCs—especially C6 and C9 unsaturated aldehydes and alcohols—play a universally critical role in conferring the ‘fresh’ flavor of horticultural crops, imparting characteristic crisp, green, or grassy sensory notes [12]. For example, in cherries, compounds such as hexanal, (*E*)-2-hexenal, and (*E*,*E*)-2,4-nonadienal are key contributors to the green and fresh aroma [13]; in cucumber, volatiles including (*E*)-2-nonenal, (*E*,*Z*)-2,6-nonadienal, and (*E*)-6-nonenal are important for the fresh flavor of the Q24 line [14]; and in watermelon, (*E*)-2-hexenal, (*E*,*Z*)-3,6-nonadien-1-ol, and (*E*,*Z*)-2,6-nonadienal are associated with a fresh and crisp flavor [15]. In litchi, various C6 and C9 VOCs such as 2-hexenal, (*E*)-2-hexenal, and nonanal have been identified across several cultivars including ‘Bingli,’ ‘Xiangmizao,’ and ‘Jingganghongnuo’ [4, 16, 17]. However, the specific C6 and C9 VOCs that define litchi’s fresh aroma, as well as the molecular mechanisms responsible for their biosynthesis, are still unknown.

Lipoxygenase (LOX; EC 1.13.11) is the rate-limiting enzyme in the biosynthesis of fatty acid-derived VOCs. As a non-heme iron-containing dioxygenase, LOX possesses a conserved His-(X)4-His-(X)4-His-(X)17-His-(X)8-His motif critical for iron binding and catalytic activity [18, 19]. Functionally, LOXs are categorized into two subgroups based on the site of oxygenation on fatty acid chains: 9-LOX, which produces C9 VOC precursors, and 13-LOX, which produces C6 VOC precursors [20]. Subsequent enzymatic steps—catalyzed by isomerases, alcohol dehydrogenases, and alcohol acyltransferases—convert the resulting hydroperoxide intermediates into aldehydes, alcohols, and esters, respectively [21, 22]. In horticultural crops, *LOX* expression is closely associated with the emission of fatty acid-derived VOCs. For instance, in pepino (*Solanum muricatum*), *SmLOXD*, *SmLOXB*, and *SmLOX5-like1* are upregulated during ripening, coinciding with increased accumulation of C6 and C9 VOCs [23]; in passion fruit (*Passiflora edulis*), the expression and enzyme activity of *PeLOX4* are positively correlated with the production of ester volatiles [24]; and in grape (*Vitis vinifera*), *VvLOXA* is essential for the biosynthesis of C6 VOCs [25]. Furthermore, emerging evidence indicates that LOXs that involved in VOC synthesis are subject to transcriptional regulation. For examples, CsCDOF1.8 directly activates *CsLOX09* to promote C9 VOC accumulation in cucumber [14]; FaLEC2 inhibits *FaLOX2* to repress VOC synthesis in strawberry [26]; and FaMYB11 activates *FaLOX5* to enhance the production of fatty acid-derived VOCs [27]. In litchi, however, the key LOXs responsible for C6 and C9 VOC biosynthesis, as well as their upstream regulators have not been identified.

In this study, we conducted a comprehensive investigation into fatty acid-derived VOCs and their regulatory mechanisms in litchi. Specifically, we profiled C6 and C9 VOCs in the arils of 24 representative cultivars to identify the key compounds determining the fresh aroma. Furthermore, we characterized *LcLOX7* as the pivotal, rate-limiting enzyme for the biosynthesis of fatty acid-derived VOCs, particularly C9 compounds. Additionally, we uncovered a novel regulatory module (LcARF17/LcRAP2-4–LcLOX7) that drives cultivar-specific aroma variation. Collectively, our findings provide a mechanistic framework for understanding the biosynthesis of fresh aroma in litchi and identify potential molecular targets for the genetic improvement of litchi flavor.

## Results

### Four key C6 and C9 volatiles define the fresh aroma of litchi arils

**Figure 1:**
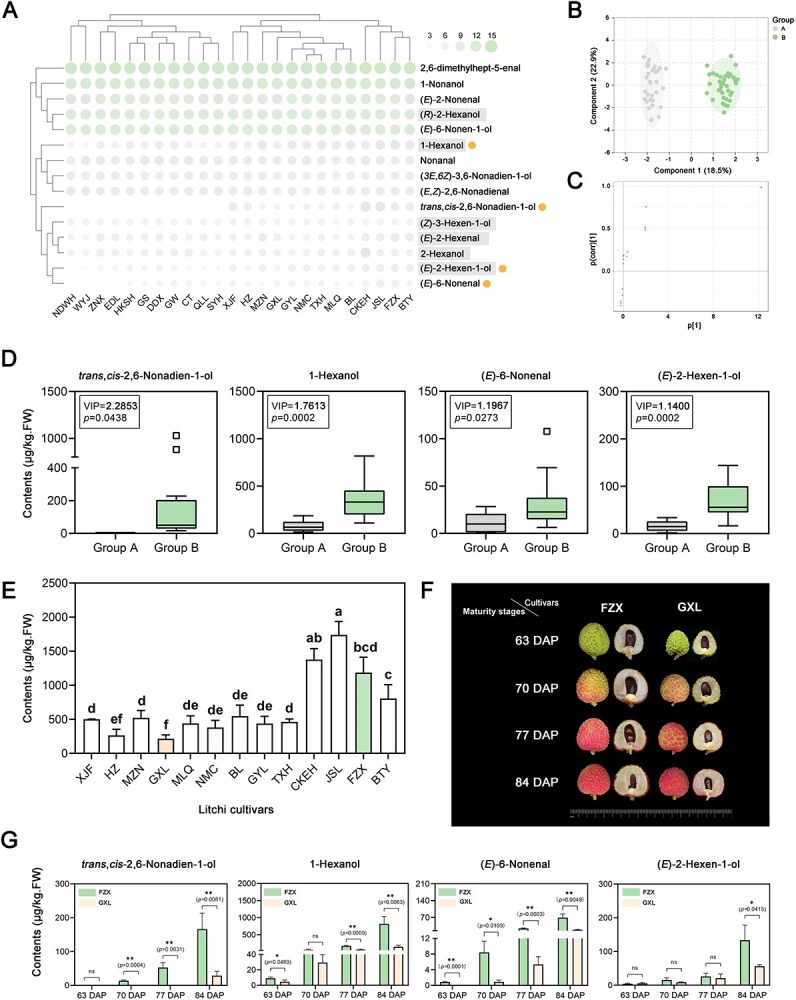
Identification of four key C6 and C9 volatiles responsible for fresh aroma profiles in litchi aril. (A) Profiling of C6 and C9 volatile compounds in litchi cultivars. A bubble chart illustrates the relative content of C6 and C9 volatile compounds detected in the arils of 24 litchi cultivars. The circle size and shading intensity are proportional to the relative content of each compound. Compounds exhibiting significant differences among cultivars are marked with distinct dots adjacent to their names. C6 volatile compounds names are highlighted with a shaded background, while those without correspond to C9 volatile compounds. (Complete relative content data are provided in Table S2.) (B) Orthogonal Partial Least Squares-Discriminant Analysis (OPLS-DA) of two groups of litchi cultivars based on C6 and C9 volatile compounds. Analyses were performed using the Metware Cloud Platform (https://cloud.metware.cn). (C) OPLS-DA S-plot based on C6 and C9 volatile compounds among two groups of 24 litchi cultivars. Green dots represent compounds with no significant intergroup differences, whereas red dots indicate compounds with a Variable Importance in Projection value greater than 1, signifying their crucial role in distinguishing the groups. (D) Boxplots illustrate the levels of key C6 and C9 volatile compounds of two litchi groups. Group A is represented by the gray box (left) and Group B by the green box (right), with squares indicating outliers. (E) Total content of four key C6 and C9 volatile compounds in Group B. A bar chart showing the total content of the four key C6 and C9 volatiles across 13 litchi cultivars in Group B. Statistically significant differences are indicated by different letters. (F) Image of fruit developmental stages of FZX and GXL litchi cultivars. DAP indicates Days After Pollination. (G) Accumulation patterns of four key C6 and C9 volatile compounds during litchi fruit maturation. Green bars (left) represent FZX cultivar, and orange bars (right) represent GXL cultivar. Statistical significance was determined by Student’s *t*-test and is indicated as follows: ^*^P < 0.05, ^**^P < 0.01, and ns for not significant.

To investigate the divergence of C6 and C9 volatiles among litchi cultivars, 24 distinct cultivars were selected for volatile profiling analysis, yielding a total of 15 volatile compounds (including six C6 and nine C9 volatiles) (Fig. 1A and Table S2). Heatmap analysis revealed a crucial differentiation factor: four specific volatiles—1-hexanol, (*E*)-2-hexen-1-ol, (*E*)-6-nonenal, and *trans*,*cis*-2,6-nonadien-1-ol—were absent or present at extremely low levels in 11 cultivars (NDWH, WYJ, ZNX, EDL, HKSH, GS, DDX, GW, CT, QLL, and SYH) compared to the other 13 cultivars (Fig. 1A, orange dots). Based on these findings, the 11 cultivars with lower levels of the aforementioned four volatiles were categorized as Group A. In contrast, the remaining 13 cultivars (XJF, HZ, MZN, GXL, GYL, NMC, TXH, MLQ, BL, CKEH, JSL, FZX, and BTY) were designated as Group B, which exhibited significantly higher abundances of the four volatiles. To validate the robustness of this grouping, Orthogonal Partial Least Squares-Discriminant Analysis (OPLS-DA) was subsequently performed, which demonstrated a clear separation between Group A (gray region) and Group B (green region) (Fig. 1B and Fig. S1). Importantly, the four volatiles previously identified in the heatmap analysis were confirmed to be responsible for this group separation (Fig. 1C). As specifically illustrated in Fig. 1D and Table S3, all four volatiles exhibited Variable Importance in Projection (VIP) values greater than 1 and showed significantly higher concentrations in Group B than in Group A. To further elucidate their contribution to litchi aroma, the Odor Activity Values (OAV) of these four key volatiles were calculated. The results showed that these volatiles, known to contribute to green and fresh aromatic notes, exhibited substantially higher OAVs in Group B compared to Group A, decisively indicating their significant role in defining the fresh flavor profile of the Group B cultivars (Table S4) [28–31]. This finding indicates their significant contribution to the fresh flavor profile of Group B cultivars.

Building on the above findings, a comparative analysis of total volatile content was conducted across the 13 cultivars in Group B to determine the optimal candidates for investigating temporal release patterns of the four key volatiles. As illustrated in Fig. 1E, cultivars JSL, CKEH, FZX, and BTY demonstrated superior volatile accumulation, whereas HZ and GXL exhibited the lowest concentrations. To maximize this variability, FZX and GXL were selected, and their fruit samples at four ripening stages were collected for subsequent investigation. Temporal analysis revealed a progressive accumulation pattern of these four key volatiles during fruit maturation (Fig. 1G and Table S5). Consistent with the total volatile content analysis (Fig. 1E), FZX emitted significantly higher levels of these volatiles than GXL across most developmental stages. Taken together, our findings confirm that the four volatiles—1-hexanol, (*E*)-2-hexen-1-ol, (*E*)-6-nonenal, and *trans*,*cis*-2,6-nonadien-1-ol, are the key compounds responsible for differentiating fresh aroma profiles in litchi fruit.

### *LcLOX7* expression is associated with the biosynthesis of key C6 and C9 volatiles in litchi aril

**Figure 2:**
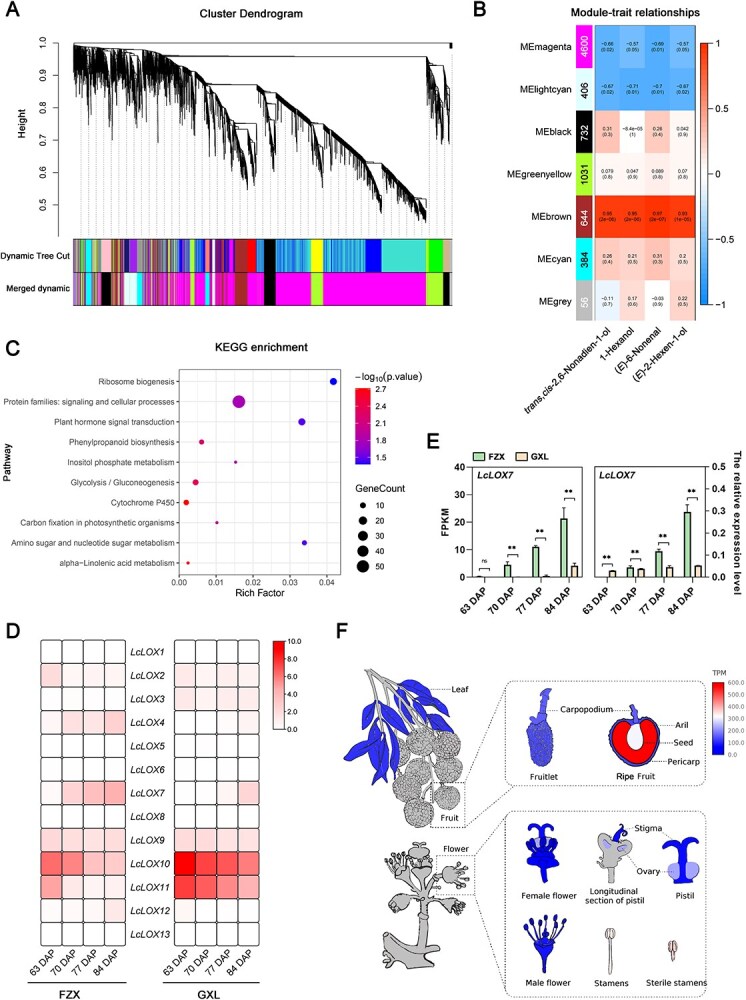
LcLOX7 is likely responsible for the biosynthesis of key C6 and C9 volatile compounds in litchi aril. (A) Weighted gene co-expression network analysis (WGCNA). A hierarchical clustering tree revealing the co-expression modules identified among the analyzed transcripts. (B) Correlation between module eigengenes and the content of key C6 and C9 volatile compounds. Each column represents the profile of a C6 and C9 volatile compound. The left panel displays the six modules, and the right panel provides a scale for module-trait correlations (ranging from −1 to 1). The intensity of shading reflects the strength of the correlation, with contrasting tones representing positive and negative correlations. Individual cells are shaded according to statistical significance and are tagged with two numbers: the upper number indicates the correlation coefficient, and the lower number indicates the *P*-value. (C) Kyoto Encyclopedia of Genes and Genomes (KEGG) enrichment analysis of the MEbrown module. Dot size corresponds to the number of enriched genes, while shading intensity reflects the statistical significance, with larger and redder dots indicating greater gene count and higher significance, respectively. (D) Expression heatmap of *LcLOX* family members in FZX and GXL cultivars. The heatmaps display the relative expression levels (log_2_ transformed) of *LcLOX* genes across four developmental stages (63, 70, 77, 84 DAP) for FZX (left) and GXL (right). The shading intensity indicates expression levels, with darker shading representing higher expression and lighter shading representing lower expression. (E) Expression patterns of *LcLOX7* during fruit development in FZX and GXL. The left box displays the transcriptome data (expressed as FPKM values), while the right box shows the RT-qPCR validation results (normalized relative expression). Error bars indicate standard deviation (SD) of three biological replicates. Statistical significance was determined by Student’s *t*-test and is indicated as follows: ^*^P < 0.05, ^**^P < 0.01, and ns for not significant. (F) Tissue-specific expression profile of *LcLOX7*. A shading gradient gradient denotes gene expression levels (low to high), with data retrieved from the Sapindaceae genome database (http://www.sapindaceae.com/index.html) [32].

To identify key *LOX* genes involved in C6 and C9 volatile biosynthesis in litchi, RNA-sequencing (RNA-seq) was performed on the arils of cultivars FZX and GXL across four developmental stages (63, 70, 77, and 84 DAP). Each sequencing library yielded ≥6.83 Gb of high-quality data. The clean reads were aligned to the reference genome of *L. chinensis* ‘Feizixiao,’ resulting in the detection of 32 656 genes, with 7853 exhibiting a Fragments Per Kilobase of exon model per Million mapped fragments (FPKM) value >4. Subsequently, weighted gene co-expression network analysis (WGCNA) was performed to explore gene-expression correlations linked to volatile biosynthesis. All detected genes were clustered into six distinct modules (excluding MEgrey as an invalid module). Each module was visualized with a unique color, and genes within the same module showed similar expression pattern (Fig. 2A). Correlation analysis further revealed that the MEbrown module showed the strongest association with the four key C6 and C9 volatiles, with Pearson’s correlation coefficients (*r*) ≥ 0.93 (*P* < 0.05) (Fig. 2B). To gain insight into the biological functions of genes in the MEbrown module, Kyoto Encyclopedia of Genes and Genomes (KEGG) enrichment analysis was performed, leading to the identification of ten significantly enriched pathways. Notably, six genes were annotated to the α-linolenic acid metabolic pathway (Fig. 2C and Table S6), and one of these genes (*LITCHI003970*) was predicted to encode a LOX family protein.

Moreover, comprehensive screening and characterization of the LOX gene family in litchi genome led to the identification of 13 LcLOX members, consisting of seven 9-LOX and six 13-LOX subfamily genes (Fig. S2A; Table S7). Among these, *LITCHI003970* (designated as *LcLOX7*) was specifically expressed in fruit, with a distinct predominance in the aril (Table S8), and its transcriptional level displayed a progressive transcriptional upregulation during fruit maturation (Fig. 2D–F). Further sequence analysis revealed that LcLOX7 contains conserved domains essential for LOX catalytic activity (Fig. S2B). Collectively, these results suggest that LcLOX7 is likely the key LOX member involved in the biosynthesis of C6 and C9 volatiles in litchi arils.

### *LcLOX7* overexpression specifically promotes the production of C9-dominant fatty acid-derived volatile

**Figure 3:**
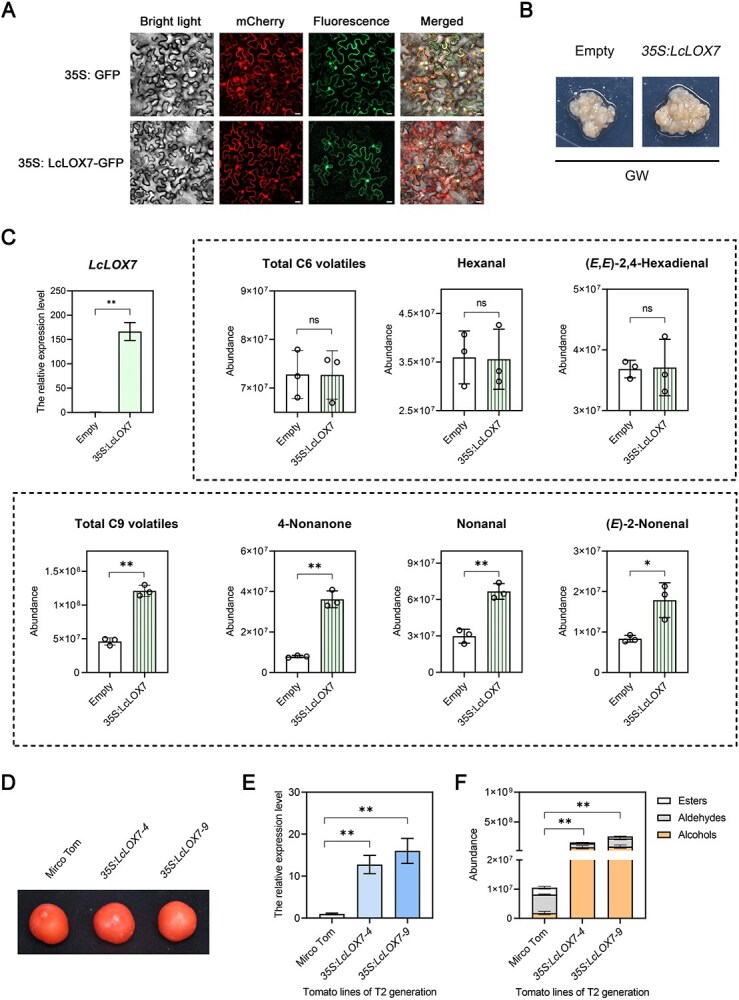
LcLOX7 promotes the production of C9-dominant fatty acid-derived volatiles. (A) Subcellular localization of LcLOX7 in tobacco leaf epidermal cells. Nuclear localization marker (At1g22590) and membrane localization marker (At5g19750) were visualized via mCherry fluorescence, LcLOX7-GFP fluorescence was captured via GFP fluorescence, alongside bright-field images. Merged images show the overlay of mCherry, GFP, and bright-field channels. Scale bars = 20 μm. (B) Image of the *LcLOX7*-overexpressed transgenic litchi callus. Callus inoculated with *Agrobacterium* carrying the empty vector served as the control. (C) *LcLOX7* expression and volatile compound detection in overexpressed litchi callus. Callus transformed with the empty vector was used as the control. In the bar chart, data outside the dashed area depict the relative expression level of *LcLOX7* in callus, as determined by RT-qPCR. The control and experimental groups are represented by white (left) and solid green bars (right), respectively. Inside the dashed area, the relative contents of C6 (upper panel) and C9 (lower panel) volatiles are presented, where white bar denote the control group (left) and green-striped bar (right) denote the *LcLOX7*-overexpression group. Statistical significance is indicated (Student’s *t*-test: ^*^P < 0.05, ^**^P < 0.01; ns = not significant). (D) Image of the *LcLOX7*-overexpressed transgenic tomato fruits. Micro-Tom was used as controls. (E) Quantitative detection of *LcLOX7*-overexpressed transgenic tomato fruits. The white (left) and blue bars (middle and right) denote the expression levels of *LcLOX7* in Micro-Tom and transgenic lines, respectively. (F) Detection of volatile compounds in *LcLOX7*-overexpressed transgenic tomato fruits. Relative contents of target volatiles are presented, with statistical significance determined by Student’s *t*-test: ^*^P < 0.05, ^**^P < 0.01.

To elucidate the role of LcLOX7 in the biosynthesis of key fresh aroma volatiles, we firstly performed subcellular localization experiments. The results demonstrated that LcLOX7 is localized to both the cell membrane and nucleus (Fig. 3A). Subsequently, *LcLOX7* was successfully overexpressed in the callus of GW cultivar via the *Agrobacterium*-mediated transformation (Fig. 3B and C). Further GC–MS analysis revealed that the production of total C9 volatiles, as well as three typical C9 volatiles (4-nonanone, nonanal, and (*E*)-2-nonenal,) were significantly higher in *LcLOX7*- overexpressed callus compared to the control. In contrast, the levels of total C6 volatiles and two typical C6 volatiles (hexanal and (*E*,*E*)-2,4-hexadienal) remained comparable between the control and transgenic callus (Fig. 3C). Moreover, *LcLOX7* was heterologously overexpressed in tomato, yielding two independent T_2_ transgenic lines. While the fruit morphology of these transgenic lines was indistinguishable from that of the wild-type (Fig. 3D and E), GC–MS analysis demonstrated that *LcLOX7*-overexpressing tomato fruits released higher levels of fatty acid-derived volatiles (including 3-hexen-1-ol, hexa-2,4-dienal, and decanal) than that in wild-type fruits (Fig. 3F and Table S9). Collectively, these results indicate that LcLOX7 plays a role in the biosynthesis of fatty acid-derived volatiles, with a pronounced effect on the production of C9-type volatile compounds.

### Transcription factors LcARF17 and LcRAP2-4 act as direct positive regulators of *LcLOX7*

**Figure 4:**
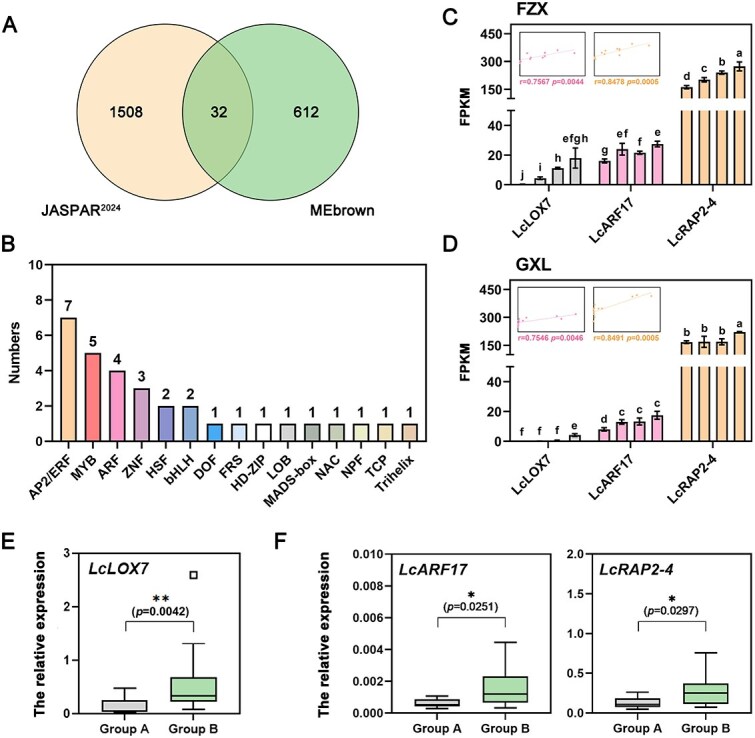
Identification of key transcription factors regulating *LcLOX7* expression. (A) Identification of key transcription factors. A venn diagram illustrates the screening of 32 key transcription factors, with the yellow (left) and green (right) circles representing candidates predicted by JASPAR^2024^ and those from the high-correlation MEbrown module in WGCNA, respectively. (B) Family classification of key transcription factors. The 32 key transcription factors are distributed across different families, with gene counts indicated above the bars. (C,D) Expression correlation analysis during fruit development. Expression profiles (FPKM) of *LcLOX7*, *LcARF17*, and *LcRAP2-4* during fruit development in two litchi cultivars (C) FZX and (D) GXL. Scatter plots depict the correlation between *LcLOX7* and *LcARF17* or *LcRAP2-4*, with Pearson correlation coefficients (r) and *p*-values indicating the strength and significance of the relationships. (E,F) Expression validation between two group litchi cultivars. RT-qPCR analysis of (E) *LcLOX7* and (F) *LcARF17* and *LcRAP2-4* in two different litchi groups. Gray boxes (left) represent Group A, and green boxes (right) represent Group B. Outliers are marked with white squares. Statistical significance determined by Student’s *t*-test: ^*^P < 0.05, ^**^P < 0.01.

Given that the expression level of *LcLOX7* gradually accumulates during the litchi fruit maturation (Fig. 2E), we hypothesized that its expression is regulated by upstream TFs. To identify candidate regulators, we extracted the 1500-bp upstream promoter sequence of *LcLOX7* and used the JASPAR^2024^ database to predicate potential binding TFs, yielding 1540 candidates. By combining these predictions with WGCNA results, 32 TFs distributed across 15 families were identified (Fig. 4A, B and Table S10). Among these, both transcriptomic and RT-qPCR analyses revealed that the expression patterns of *LcARF17* and *LcRAP2-4* exhibited a strong positive correlation with *LcLOX7* expression (Fig. 4C, D, Fig. S3 and Table S10). Importantly, significantly higher expression of *LcARF17* and *LcRAP2-4* was observed in Group B (high fresh aroma cultivars) compared to Group A (low fresh aroma cultivars), consistent with the expression pattern of *LcLOX7* and the accumulation profiles of key volatile compounds (Figs 1D and 4E, F). Taken together, these results indicate that LcARF17 and LcRAP2-4 are potential key regulators of *LcLOX7* in the biosynthesis of fresh aroma volatiles.

**Figure 5:**
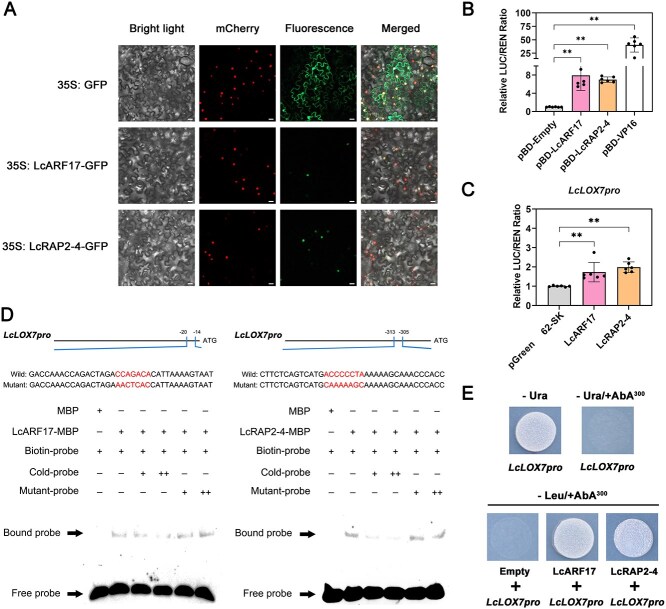
LcARF17 and LcRAP2-4 directly activate *LcLOX7* transcription. (A) Subcellular localization of LcARF17 and LcRAP2-4 in tobacco leaf epidermal cells. The nuclear localization marker (At1g22590) was detected via mCherry fluorescence, GFP signals were visualized via GFP fluorescence, and a bright-field image was also captured. Merged images show the overlays of mCherry, GFP, and bright-field signals. Scale bars = 20 μm. (B) Transcriptional activation assay of LcARF17 and LcRAP2-4 in tobacco leaves. Bar chart quantifying the inherent transcriptional activation activity of LcARF17 and LcRAP2-4. pBD-Empty and pBD-VP16 were used as negative and positive control, respectively. (C) Transient dual-luciferase assays showing activation of *LcLOX7* promoter by LcARF17 and LcRAP2-4. Bar chart quantifying the enhanced activity of the *LcLOX7* promoter when co-expressed with LcARF17 and LcRAP2-4. pGreen 62-SK was used as the negative control. Data are presented as mean ± SD. Statistical significance determined by Student’s *t*-test: ^*^P < 0.05, ^**^P < 0.01. (D) *In vitro* binding of LcARF17 and LcRAP2-4 to the *LcLOX7* promoter was demonstrated by electrophoretic mobility shift assay (EMSA). Arrows highlight shifted bands indicating DNA-protein complex and free probe formations. ‘−’ and ‘+’ represent absence or presence of the respective component; ‘++’ indicates the increasing amounts of unlabeled cold probes (for competition) or mutant probes. MBP-tagged protein alone served as a negative control. (E) Yeast one-hybrid analysis of LcARF17 and LcRAP2-4 binding to the promoter of *LcLOX7*. No basal activities of *LcLOX7* promoter were detected in yeast grown on SD/-Ura medium with 300 ng/ml aureobasidin A (AbA). The interaction was evaluated based on the growth conditions of transformed yeast on SD/-Leu medium containing 300 ng/ml AbA.

To validate the binding relationship between LcARF17/LcRAP2-4 and *LcLOX7* promoter, a series of complementary *in vivo* and *in vitro* experiments were performed. Subcellular localization assays first confirmed that both LcARF17 and LcRAP2-4 are localized to the nucleus (Fig. 5A), consistent with their predicted role as TFs. The dual-luciferase reporter assay was then utilized to evaluate their transcriptional activation capabilities (Fig. S4). Transient expression of pBD-LcARF17 and pBD-LcRAP2-4 in tobacco leaves resulted in significant increases in the LUC/REN ratio (7.92-fold and 7-fold, respectively) (Fig. 5B). Furthermore, co-expression of LcARF17 or LcRAP2-4 with the *LcLOX7pro*:LUC construct enhanced *LcLOX7* promoter activity by 1.73-fold and 2-fold, respectively (Fig. 5C). Crucially, Electrophoretic Mobility Shift Assay (EMSA) results demonstrated the direct binding of LcARF17 and LcRAP2-4 to labeled probes derived from *LcLOX7* promoter, forming DNA-protein complexes that were effectively competed by cold probes, but not by mutant probes (Fig. 5D). Yeast one-hybrid assays further corroborated these interactions, as yeast cells co-transformed with LcARF17 or LcRAP2-4 and the *LcLOX7* promoter grew normally on SD/-Leu medium containing Aureobasidin A (Fig. 5E), whereas controls did not. Collectively, these complementary findings strongly suggest that both LcARF17 and LcRAP2-4 act as direct positive regulators of *LcLOX7* by binding to its promoter.

### The regulatory modules LcARF17/LcRAP2-4-LcLOX7 play a role in C9 volatile emission

**Figure 6:**
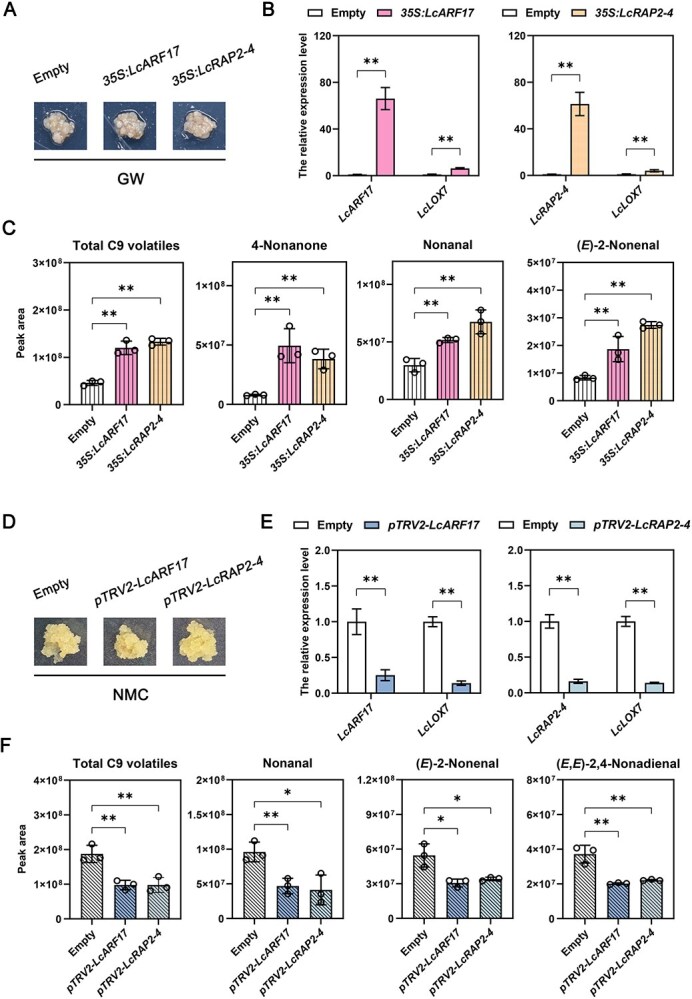
LcARF17 and LcRAP2-4 promote LcLOX7-mediated C9 volatile release in litchi callus. (A) Image of *Agrobacterium*-infected litchi callus (GW cultivar). Callus transfected with the empty vector served as the control. (B) Expression levels of gene-overexpressed litchi callus. Control groups are represented by unfilled bars, while LcARF17- and LcRAP2-4-overexpression groups are represented by shaded bars. (C) Abundance of C9 volatiles in *LcARF17*/*LcRAP2-4*-overexpressed litchi callus. For each compound, bars from left to right represent the control (Empty), LcARF17-overexpression, and LcRAP2-4-overexpression groups. (D) Image of *Agrobacterium*-infected litchi callus (NMC cultivar). Callus transfected with the empty vector served as the control. (E) Expression levels of gene-silenced litchi callus. Control groups are represented by unfilled bars, while LcARF17- and LcRAP2-4-silenced groups are represented by shaded bars. (F) Abundance of C9 volatiles in LcARF17/LcRAP2-4-silenced litchi callus. For each compound, bars from left to right represent the control (Empty), LcARF17-silenced, and LcRAP2-4-silenced groups. Statistical significance was determined by Student’s *t*-test and indicated as follows: ^*^P < 0.05, ^**^P < 0.01, and ns for not significant.

Given the direct transcriptional activation of *LcLOX7* by LcARF17 and LcRAP2-4, we proposed that these TFs play a role in the biosynthesis of volatile compounds. To test this hypothesis, *LcARF17* and *LcRAP2-4* were overexpressed in the low-fresh aroma cultivar GW callus (Fig. 6A), while they were silenced in the high-fresh aroma cultivar NMC callus (Fig. 6D). In overexpressed callus lines, the expression levels of *LcARF17* and *LcRAP2-4* was elevated by 66.08-fold and 61.38-fold, respectively. This marked upregulation was accompanied by a subsequent 6.23-fold and 4.02-fold increase in *LcLOX7* gene expression in *LcARF17*- and *LcRAP2-4-*overexpressed callus, respectively (Fig. 6B). Conversely, silencing of *LcARF17* and *LcRAP2-4* in NMC callus drastically decreased their own expression (by 3.97-fold and 6.21-fold, respectively) and led to a corresponding decline in *LcLOX7* transcripts (by 7.12-fold and 25.23-fold) (Fig. 6E). Together, these findings indicate that LcARF17 and LcRAP2-4 can promote *LcLOX7* expression in litchi callus.

Furthermore, GC–MS analysis demonstrated that the overexpression of either *LcARF17* or *LcRAP2-4* significantly enhanced the abundance of three C9 volatiles (4-nonanone, nonanal, and (*E*)-2-nonenal) in the transformed callus (Fig. 6C and Table S11). Notably, no significant effect was observed on total C6 volatile content, with the sole exception of (*E*,*E*)-2,4-hexadienal which was significantly higher only in *LcARF17*-overexpressed callus (Fig. S5A and Table S11). Conversely, silencing *LcARF17* or *LcRAP2-4* in NMC callus resulted in a marked reduction in C9 volatile emissions (Fig. 6F and Table S12), while again showing no significant effect on total C6 volatile content (Fig. S5B and Table S12). Collectively, these results strongly suggest that LcARF17 and LcRAP2-4 enhance the production of C9 volatiles in litchi callus via promoting *LcLOX7* expression.

## Discussion

Previous studies have established that C6 and C9 VOCs are pivotal for the formation of fresh aroma in horticultural crops, with their quantitative variations directly dictating the intensity of fresh fragrance [33, 34]. However, these studies focused on a limited number of cultivars, failing to identify core aroma-determining C6 and C9 VOCs for defining distinguishing cultivar-specific aroma profiles. Here, we address this gap by not only identified four key C6 and C9 VOCs as determinants of fresh aroma variation in litchi aril but also elucidated two transcriptional regulatory modules that govern their biosynthesis.

**Figure 7:**
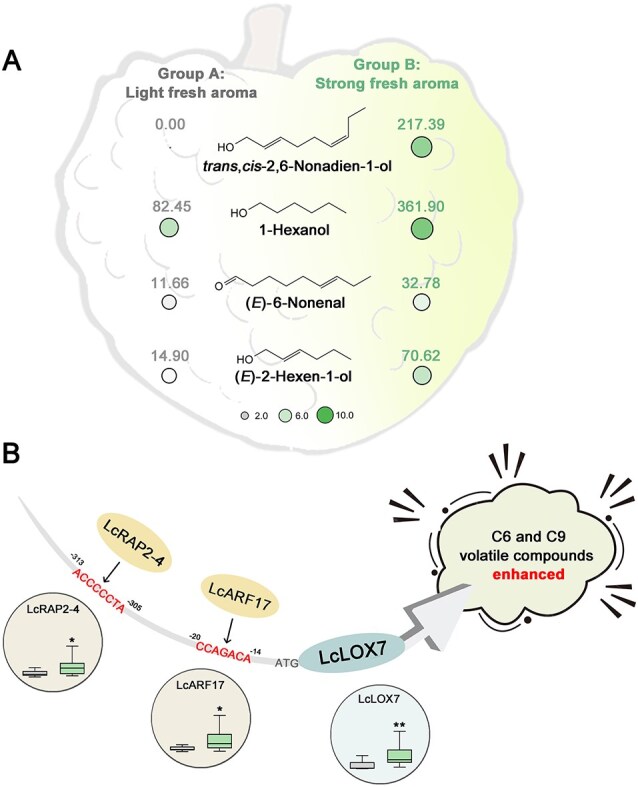
Variation in fresh aroma volatiles between two distinct groups litchi cultivars and a proposed working model of LcARF17/RARP2-4-LcLOX7 in regulating fresh aroma biosynthesis in litchi. (A) Variation in four key fresh aroma volatiles between Group A and Group B litchi cultivars. Circle size and shading intensity represent the relative abundance of each compound, with numerical values indicated. (B) Proposed working model for fresh aroma regulation during litchi fruit maturation. LcARF17 and LcRAP2-4 independently activate *LcLOX7* expression by binding to distinct promoter motifs. This transcriptional activation specifically enhances the biosynthesis of fresh aroma volatiles, particularly C9 compounds. The simplified box plots in circles represent volatile levels, with gray (left) and green (right) boxes indicating Group A (low-aroma) and Group B (high-aroma) cultivars, respectively.

To identify the key VOCs that determine litchi fresh aroma, we conducted a comprehensive profiling of C6 and C9 VOCs across 24 representative litchi cultivars (Fig. 1A, Table S2), a scale that exceeds all previous litchi aroma studies. Our analysis identified four key VOCs: *trans,cis*-2,6-nonadien-1-ol, 1-hexanol, (*E*)-6-nonenal, and (*E*)-2-hexen-1-ol. These compounds are functionally critical as they not only accumulated progressively during fruit maturation but also effectively discriminated cultivars with varying fresh aroma intensities (Fig. 7A). Each compound contributes to distinct olfactory characteristics: *trans,cis*-2,6-nonadien-1-ol and (*E*)-6-nonenal impart cucumber/melon-like green notes, 1-hexanol contributes herbaceous green aroma, and (*E*)-2-hexen-1-ol adds fruity-green nuances [35–38]. Their significantly higher OAVs observed in aromatic cultivars (Table S4) further confirm their functional relevance to fresh aroma perception. Collectively, these findings fill a critical knowledge gap by identifying the first set of universal biomarkers for litchi fresh aroma, offering a practical and precise basis for future flavor-oriented breeding programs and quality assessment.

LOX serves as the rate-limiting enzyme in fatty acid-derived VOC biosynthesis, where its expression and enzymatic activity directly govern fresh aroma emission in fruits. For instance, overexpression of *Mi9LOX* in mango enhanced lactone accumulation [39], while silencing *CsLOX9* in cucumber drastically reduced C9 aldehyde and alcohol levels [14]. In the present study, WGCNA and KEGG enrichment analysis consistently identified *LcLOX7*, a 9-LOX subfamily member, as a core gene strongly correlating with the four key fresh-aroma VOCs (Fig. 2). Functional validation indicated that LcLOX7 plays a specific role in fatty acid-derived VOC biosynthesis, exhibiting a pronounced preference for C9 VOC production (Fig. 3). However, interpreting the precise *in vivo* contribution of LcLOX7 requires consideration of experimental limitation and gene family complexity. Our functional validation was conducted in heterologous (tomato) and litchi callus systems. Both systems lack the complex metabolic microenvironment of intact fruit tissues, potentially failing to fully replicate the native VOC profile of litchi. Furthermore, LOX family members commonly exhibit functional redundancy in plant. For instance, in pepino, three core *LOX* genes (*SmLOXD*, *SmLOXB*, and *SmLOX5-like1*) are collectively associated with LOX-derived aroma biosynthesis [23]; similarly, both FaLOX2 and FaLOX5 function redundantly in contributing to fatty acid-derived VOC production in strawberry [26, 27]. In the context of litchi, we identified 13 LOX members exhibiting widespread tissues expression (Table S8). While *LcLOX7* was the strongest candidate, showing the highest expression specifically in the aril and a progressive transcriptional upregulation concomitant with fruit maturation (Fig. 2E and F), other LOX members cannot be discounted. For examples, *LcLOX4* also showed ripening-associated accumulation (albeit with low aril expression), and *LcLOX8* exhibited high pericarp expression with non-negligible levels in the aril. These tissue-specific co-expression patterns suggest that while LcLOX7 serves as the primary driver of C9 aroma in the litchi aril, the combined or sequential actions of multiple LOX members may collectively shape the overall aroma profile of litchi. Therefore, further studies employing targeted in planta manipulation of other LOX family members are essential to fully elucidate their individual and synergistic contributions to litchi fresh aroma formation.

Although ARFs and AP2/ERFs TFs have been implicated in regulating fruit aroma in horticultural crops [40–42], their precise molecular mechanisms remain largely elusive. In this study, we uncovered a novel regulatory pathway connecting LcARF17 and LcRAP2-4 with the LcLOX7-mediated biosynthesis of fresh aroma compounds in litchi. Integrated analysis and functional assays demonstrated that LcARF17 and LcRAP2-4 directly bind to the *LcLOX7* promoter and activate its transcription (Fig. 5B-E), and functional validation in litchi callus confirmed that their manipulation robustly affects *LcLOX7* expression and corresponding C9 volatile emission (Fig. 7B), thereby revealing the LcARF17/LcRAP2-4-LcLOX7 regulatory modules that positively regulate the biosynthesis of fresh aroma in litchi. Furthermore, we propose that LcARF17 and LcRAP2-4 might function independently in this process based on following evidence: (i) the absence of *in vivo* and *in vitro* protein interaction between LcARF17 and LcRAP2-4 (Fig. S6A and B); (ii) the lack of mutual transcriptional cross-regulation (Fig. S6C and D); (iii) both LcARF17 and LcRAP2-4 possess comparable intrinsic regulatory capability over the *LcLOX7* promoter and the final C9 volatile output (Fig. 5C). We therefore conclude that LcARF17 and LcRAP2-4 independently and comparably promote C9 volatile biosynthesis in litchi by activating *LcLOX7* expression.

In conclusion, we identified four C6 and C9 VOCs—*trans,cis*-2,6-nonadien-1-ol, 1-hexanol, (*E*)-6-nonenal, and (*E*)-2-hexen-1-ol—as the key determinants of fresh aroma variation in litchi fruit. Based on their abundance, litchi cultivars can be classified into two distinct groups: light-aroma cultivars (Group A), characterized by low levels of these four VOCs, and strong-aroma cultivars (Group B), possessing significantly higher concentrations of these volatiles. We further elucidated a novel transcriptional regulatory module on LcARF17/LcRAP2-4–LcLOX7 that govern fresh aroma formation in litchi fruit. Specifically, LcARF17 and LcRAP2-4 independently activate *LcLOX7* expression by binding to distinct promoter motifs, thereby promoting the biosynthesis of fresh aroma volatiles, particularly C9 compounds. Collectively, our findings unravel a previously uncharacterized molecular pathway governing fatty acid-derived aroma formation in litchi, providing new insights into the transcriptional regulation of fruit flavor and a robust genetic basis for aroma-oriented breeding strategies.

## Materials and methods

### Plant materials

In the summer of 2022, a total of 24 litchi cultivars were selected for sampling from the Dongguan Botanical Garden (Guangdong Province, China), including ‘Baitangying’ (BTY), ‘Bingli’ (BL), ‘Caikenerhao’ (CKEH), ‘Chunteng’ (CT), ‘Dadingxiang’ (DDX), ‘Edanli’ (EDL), ‘Feizixiao’ (FZX), ‘Guanxiangli’ (GXL), ‘Guanyinlv’ (GYL), ‘Guishuang’ (GS), ‘Guiwei’ (GW), ‘Haikensihao’ (HKSH), ‘Huaizhi’ (HZ), ‘Jiangshalan’ (JSL), ‘Meiliqiu’ (MLQ), ‘Miaozhongnuo’ (MZN), ‘Naidaowuhe’ (NDWH), ‘Nuomici’ (NMC), ‘Qinglongli’ (QLL), ‘Sanyuehong’ (SYH), ‘Tangxiahong’ (TXH), ‘Wuyejiu’ (WYJ), ‘Xianjingfeng’ (XJF), and ‘Ziniangxi’ (ZNX). During the cultivation period, all litchi cultivars were grown under identical cultivation regimes, including uniform irrigation, fertilization, and pest management practices. Despite natural variation leading to distinct flowering dates among cultivars, their fruit maturity periods were comparable. Therefore, fruit arils (edible pulp) were harvested at 84 days after pollination (DAP) for each cultivar; sampling dates were adjusted according to individually recorded pollination events to maintain synchronized developmental stages. Notably, all pollination occurred naturally via insect vectors, with no manual intervention. For each cultivar, samples were collected from three individual trees, each serving as an independent biological replicate. Specifically, each individual sample was subjected to separate volatile compound extraction and GC–MS analysis to ensure data reliability. Immediately after collection, samples were snap-froze in liquid nitrogen and stored at −80°C for subsequent experiments.

### GC–MS analysis

Before headspace adsorption, quick-frozen litchi arils were ground. An aliquot of 500 mg sample, 2 ml of saturated NaCl solution, and 0.5 μg of internal standard (3-hexanone) were added to a 20 mlheadspace vial. The prepared samples were incubated at 60°C with shaking for 5 min. Subsequently, a 120 μm DVB/CWR/PDMS fiber (Agilent) was inserted into the headspace vial for 15 min to adsorb volatile compounds. The trapped volatile compounds were then thermally desorbed and transferred to an Agilent 8890-7000D GC–MS/MS system equipped with a DB-5MS capillary column (0.25 mm inner diameter, 30 m length, 0.25 μm film thickness). The GC oven temperature program was set as follows: initial isothermal hold at 40°C for 3.5 min, followed by an increase to 100°C at 10°C/min, a further ramp to 180°C at 7°C/min, and a final elevation to 280°C at 25°C/min with a 5 min hold. The MS conditions were as follows: Electron Ionization (EI) source, ion source temperature 230°C, quadrupole temperature 150°C, MS interface temperature 280°C, electron energy 70 eV, scanning mode: Selected Ion Monitoring (SIM), and precise scanning of qualitative and quantitative ions (GB 23200.8-2016). Compounds were identified by comparing their mass spectra with those in the NIST 2011 mass spectral library.

The content of volatile terpenoid compounds was semi-quantified using the volatile internal standard method. The concentration of each compound is expressed as micrograms per kilogram of fresh weight (μg/kg · FW) and calculated using the following formula:


\begin{align*} &\mathrm{Content}\ \left(\mathrm{\mu} \mathrm{g}/\mathrm{kg}\cdotp \mathrm{FW}\right)\\ &\quad=\frac{\mathrm{Target}\ \mathrm{Peak}\ \mathrm{Area}\times 0.5}{\mathrm{Internal}\ \mathrm{Standard}\ \mathrm{Peak}\ \mathrm{Area}\times \mathrm{Sample}\ \mathrm{Weight}\ \left(\mathrm{kg}\right)} \end{align*}

### RNA-seq sequencing and data analysis

RNA-seq was performed on aril tissues of the FZX and GXL cultivars across four developmental stages (63, 70, 77, and 84 DAP), with three biological replicates for each sample. Raw sequencing data were first subjected to quality control using fastp (v0.19.3) with default parameters to trim low-quality bases and remove adapter sequence. High-quality RNA-seq reads were then aligned to the litchi reference genome using Hisat2 (v2.1.0) with default settings. The expression levels of transcripts in each sample were determined by calculating FPKM values using featureCounts (v1.6.1). Finally, genes exhibiting an FPKM value greater than 4 across all samples were selected for constructing the co-expression network using the WGCNA package (v1.73) in R.

### RT-qPCR analysis

Total RNA was extracted from litchi arils using the RNAprep Pure Plant Plus kit (Tiangen). RT-qPCR was performed using the Hieff qPCR SYBR Green Master Mix (YEASEN) on an ABI7500 Real-Time PCR System. All RT-qPCR analyses were performed in triplicate (three biological replicates). Relative gene expression levels were calculated using the 2^−△△Ct^ method. *LcEF-1α* was employed as the internal reference gene for litchi [43], while *SlUBI* served as the internal reference gene for normalizing expression levels in tomato [44]. All primers used in the analysis are listed in Table S1.

### Weighted correlation network analysis and gene network visualization

Following the removal of undetectable or low-expression genes (those with a mean FPKM <4), co-expression network modules were constructed using the WGCNA package in R [45]. Modules were generated using the automatic network construction function (blockwiseModules) with default settings, after which initial clusters were merged based on their module eigengenes. The eigengene value for each module was subsequently calculated to investigate associations with four key volatile compounds present in litchi arils. The selection of TFs was a two-step process: candidates were initially guided by predictions from the JASPAR^2024^ database [46], and then cross-referenced with the highly correlated WGCNA modules containing structural genes to determine the final potential regulatory targets.

### Gene identification and sequence analysis

To identify the LOX gene family members in litchi, two complementary search strategies were employed. First, six *Arabidopsis thaliana* LOX family members [47] were used as bait queries to perform a BLAST search against the assembled litchi genome database. Second, a genome-wide search for litchi LOX genes was conducted using HMMER with the Pfam numbers PF00305 [48]. Multiple sequence alignment of the identified LOX proteins was performed using MUSLE [49]. Phylogenetic tree construction was carried out using IQ-TREE [50], and the resulting tree was visualized and annotated with iTOL [51]. Finally, conserved domain analysis was constructed and visualized using TBtools [52].

### Gene cloning, recovery, and ligation

All gene primers used in the experiments are listed in Table S1. Prior to cloning, Total RNA was extracted from litchi arils using the RNAprep Pure Plant Kit (Tiangen). The extracted RNA was then reverse-transcribed into cDNA using the One-Step gDNA Removal and cDNA Synthesis SuperMix (TransGen), which served as the template for target gene amplification. Gene cloning was performed using ApexHF HS DNA Polymerase FS (Accurate Biology) with the following PCR protocol: initial denaturation at 95°C for 5 min, followed by 32 cycles of denaturation at 95°C for 30 s, annealing at 60°C for 30 s, and extension at 72°C for 1 min, with a final extension at 72°C for 5 min. The amplified DNA fragments were purified using the DNA Gel Extraction Kit (Tsingke). The fragments were subsequently ligated into the target vector using the ClonExpress II One Step Cloning Kit (Vazyme).

### Subcellular localization analysis

The coding sequences of *LcLOX7*, *LcARF17*, and *LcRAP2-4* were cloned and individually inserted into the pCAMBIA1302 vector to fuse with the GFP reporter gene (primers are listed in Table S1). *Agrobacterium* strain GV3101 cells harboring pCAMBIA1302-*LcLOX7*-GFP, pCAMBIA1302-*LcARF17*-GFP, pCAMBIA1302-*LcRAP2-4*-GFP, or empty pCAMBIA1302-GFP (used as a control), were transiently infiltrated into the leaves of 4-week-old *Nicotiana benthamiana* plants, following the procedure described previously [53]. GFP signals were detected 48 to 72 hours post-infiltration using a Confocal Laser Scanning Microscope (LSM 7 DUO; Zeiss) at an excitation wavelength of 488 nm.

### Dual-luciferase reporter assay

To investigate the intrinsic transcriptional activity of the TFs, the coding sequences of *LcARF17* and *LcRAP2-4* were individually fused to the reporter vector constructed based on the pBD vector [54], which served as the effector plasmids. To study the specific binding and activation of LcARF17 and LcRAP2-4 to the *LcLOX7*, the *LcLOX7* promoter was inserted into the pGreenII 0800-LUC vector (reporter construct). *LcARF17* and *LcRAP2-4* were separately inserted into the pGreenII 62-SK vector (effector constructs). Each pair of effector and reporter plasmids was co-transformed into tobacco leaves via *Agrobacterium*-mediated transient expression, as described previously [55]. After two days of incubation, LUC (Firefly Luciferase) and REN (Renilla Luciferase) luciferase activities were detected using the Dual-luciferase assay reagents (YEASEN). The transcriptional activity was calculated as the ratio of LUC to REN (LUC/REN). For each plasmid pair, six independent replicates were assessed at least. Primers used for vector construction were listed in Table S1.

### Electrophoretic mobility shift assays

The coding sequence of *LcARF17* and *LcRAP2-4* were cloned and inserted into pMAL-c2 vector. These constructs were subsequently introduced into *Escherichia. coil* strain BM Rosetta (DE3). Following induction with 1 mM isopropyl thio-β-D-galactoside (IPTG) at 16°C for 20 h, the MBP-LcARF17 and MBP-LcRAP2-4 fusion proteins were purified using MBP Sep Dextrin Agarose Resin 6FF (YEASEN). Biotin-labeled probes (3′-end labeled), containing the predicted binding sites derived from the *LcLOX7* promoter, were synthesized. Unlabeled identical sequences (cold probes) and mutant probes were synthesized to serve as competitors. The synthesized probes were incubated with the corresponding purified MBP-LcARF17 or MBP-LcRAP2-4 fusion proteins. The DNA-binding assays were then performed using the Lightshift Chemiluminescent EMSA Kit (Thermo Fisher Scientific). The resulting signals, indicating the formation of DNA-protein complexes, were imaged on a ChemiDoc MP Imaging System (Bio-Rad).

### Yeast one-hybrid assay

The yeast one-hybrid assays were performed following the method described by Ma *et al*. [56]. For prey vector construction, the coding sequences of *LcARF17* and *LcRAP2-4* were amplified and inserted into the pGADT7 vector, respectively. For bait vector construction, the *LcLOX7* promoter fragment was cloned into the pAbAi vector. Subsequently, the yeast one-hybrid assay was conducted using the Matchmaker™ Gold Yeast One-Hybrid Library Screening System (BD Clontech). The formation of the DNA-protein interaction was assessed based on the growth of the transformed yeast on SD/-Leu medium containing the antibiotic Aureobasidin A.

### Transformation experiment of litchi callus

The target gene was stably transformed into litchi calli following the culture and *Agrobacterium*-mediated transformation method described by Qin *et al*. [57]. Four weeks after transformation, the successfully transformed samples were subjected to RT-qPCR analysis to confirm target gene expression levels. For the detection of volatile compounds, 1 g of callus was accurately weighed and placed in a 20 ml headspace bottle. The bottle was sealed for 24 h to allow for volatile compound accumulation in the headspace. Volatile compounds were then adsorbed using a solid-phase Microextraction (SPME) fiber for 12 h before subsequent analysis by GC–MS. The detailed procedures and methods for the SPME-GC–MS analysis followed our previously established protocol.

### Heterologous transformation of tomato

The coding sequence of *LcLOX7* was amplified and inserted into the pCAMBIA1302 vector (Table S1), placing it under the control of the constitutive 35S cauliflower mosaic virus promoter for high-level expression. The constructed vector was transformed into competent cells of *Agrobacterium tumefaciens* (strain GV3101). Tomato cotyledons were subsequently transformed using the established leaf disk method [58]. The resulting T_1_ generation of transgenic tomato plants, along with wild-type (WT) control plants, were grown to maturity. Volatile compounds in the fruits were then analyzed using GC–MS to assess the functional impact *LcLOX7* overexpression on the fruit’s volatile profile.

### Bimolecular fluorescence complementation assay

The full-length or truncated cDNA sequences of LcARF17 and LcRAP2-4 were cloned into pSPYNE or pSPYCE vectors to construct fusions expressing the N-terminal (YFP^N^) and C-terminal (YFP^C^) fragments of yellow fluorescent protein (YFP), respectively (Table S1). The resulting fusion expression vectors were transformed into *Agrobacterium* (strain GV3101). The *Agrobacterium* mixture, containing two different plasmid constructs of equal volume, was instantly transformed into *N. benthamiana* leaves, as described by Schutze *et al*. [59]. The infiltrated *N. benthamiana* plants were incubated at 26°C under an 8-h light/16-h dark photoperiod for 3 days. YFP Fluorescence, indicating a successful protein–protein interaction and the reconstitution of the YFP signal, was observed at 488 nm using a Confocal Laser Scanning Microscope (LSM 7 DUO; Zeiss) in the infiltrated leaf areas.

### Yeast two hybrid assay

The full coding sequence of *LcRAP2-4* was inserted into the pGBKT7 vector, resulting in the constructs BD-LcRAP2-4. Similarly, the full-length coding sequence of LcARF17 was cloned into pGADT7 vector, creating AD-LcARF17. These complementary pairs of plasmids were co-transformed into Yeast Two Hybrid Gold yeast cells. The transformed cells were cultured at 30°C on the stringent selective medium: SD/-Leu/-Trp/-His/-Ade. Potential protein–protein interactions were evaluated based on the subsequent yeast growth and α-galactosidase activity on this selective medium. Detailed experimental procedures followed the methodology described by wang *et al*. [60].

### Data analysis

Experimental data are presented as the means ± standard deviation from three or six independent biological replicates to ensure statistical robustness. Statistical significance was determined by Student’s *t*-test, as appropriate. All primers used in this study are listed in Table S1.

## Supplementary Material

Web_Material_uhag010

## Data Availability

All data generated or analyzed during this study are included in this published article and its supplementary information files.
