# Supplementary material for: Involvement of the LcARF17- and LcRAP2-4-LcLOX7 regulatory modules in the biosynthesis of fresh aroma in litchi aril
Source: Hortic Res. 2026 Jan 9;13(4):uhag010. doi: 10.1093/hr/uhag010 (PMC13095353; doi:10.1093/hr/uhag010)
Supplement: Web_Material_uhag010 [file web_material_uhag010.zip › Supplementary Figure .pdf]

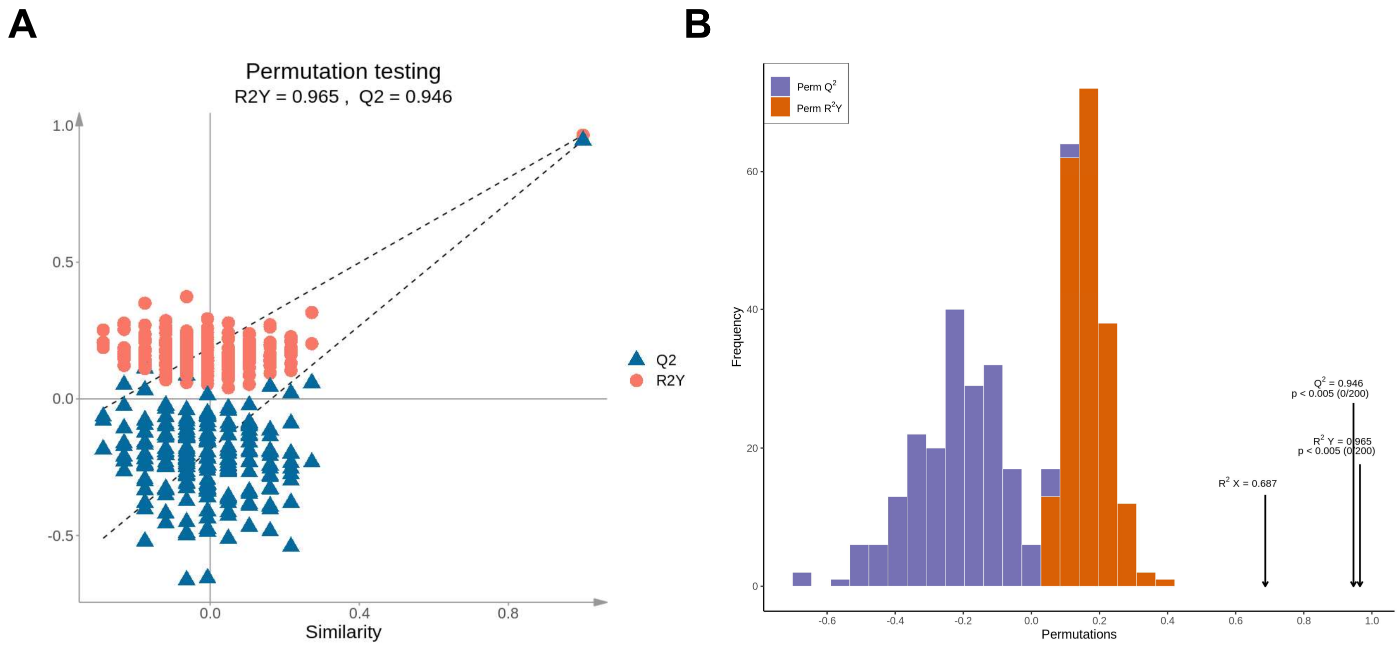

Figure S1. Credibility analysis of the OPLS-DA model. A) OPLS-DA permutation test plot. The permutation test model randomly shuffles the group labels (Y-variable) of the experimental and control groups, then repeatedly (typically n=200 times) constructs corresponding OPLS-DA models to obtain the R2Y and Q2 values of the randomized models. Higher values indicate better predictive performance and stronger explanatory power of the model. B) OPLS-DA model validation plot. The model's predictive parameters include R^2X, R^2Y, and Q^2. Here, R^2X and R^2Y represent the model's explanatory power for the X and Y matrices, respectively, while Q^2 reflects its predictive capability. The closer these three metrics are to 1, the more stable and reliable the model is. A model is considered valid when $Q^2 > 0.5$ and excellent when $Q^2 > 0.9$.

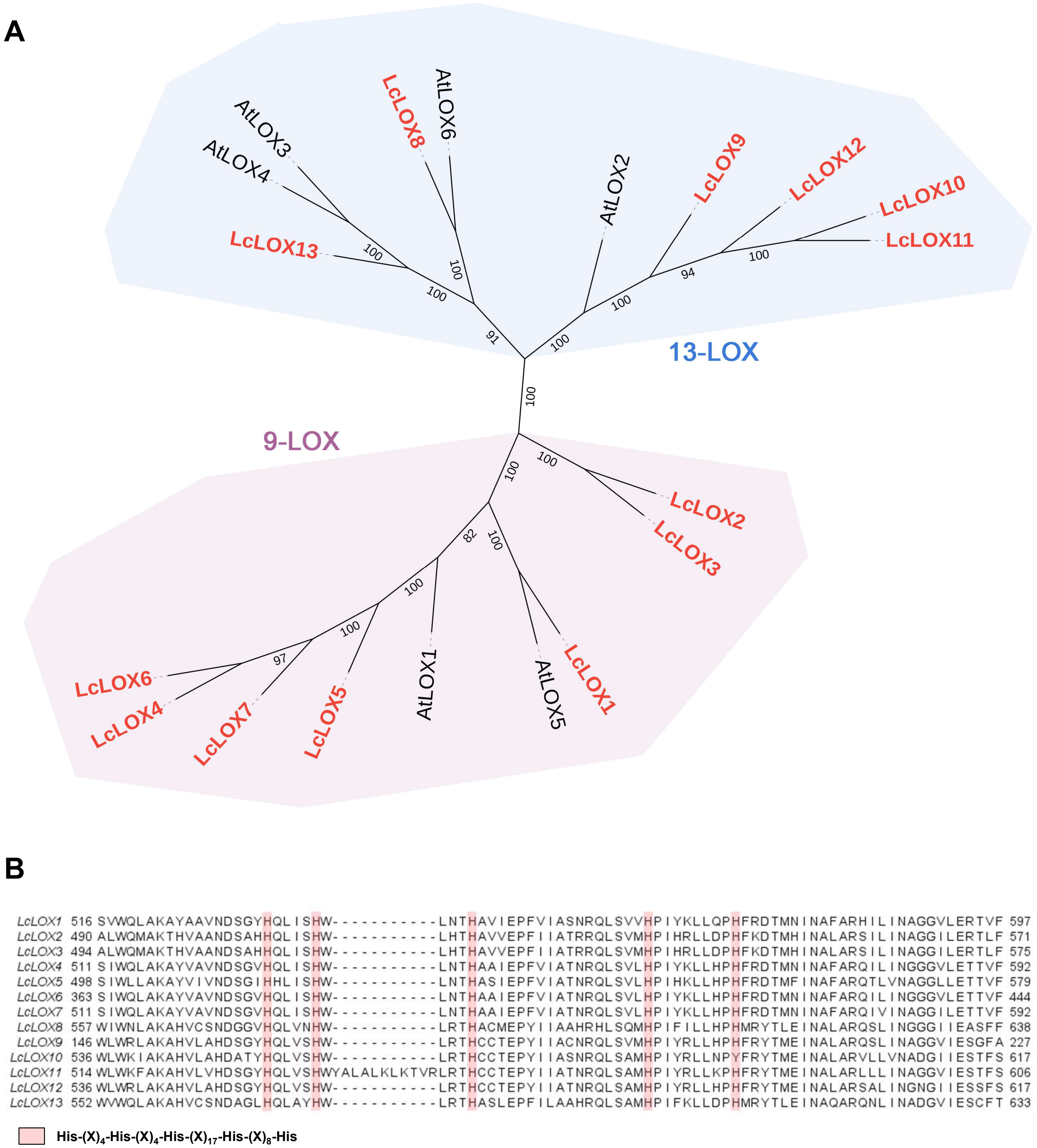

Figure S2. Identification and analysis of the LcLOXs family. A) Phylogenetic tree of LOX family members in litchi and *Arabidopsis*. Litchi LOX family members are represented in red, while *Arabidopsis* LOX family members are represented in black. B) Conserved domain analysis of LcLOX proteins. Conserved domains—His-$(X)_4$-His-$(X)_4$-His-$(X)_{17}$-His-$(X)_8$-His—are highlighted. Numbers represent the positions of the last residue in each line.

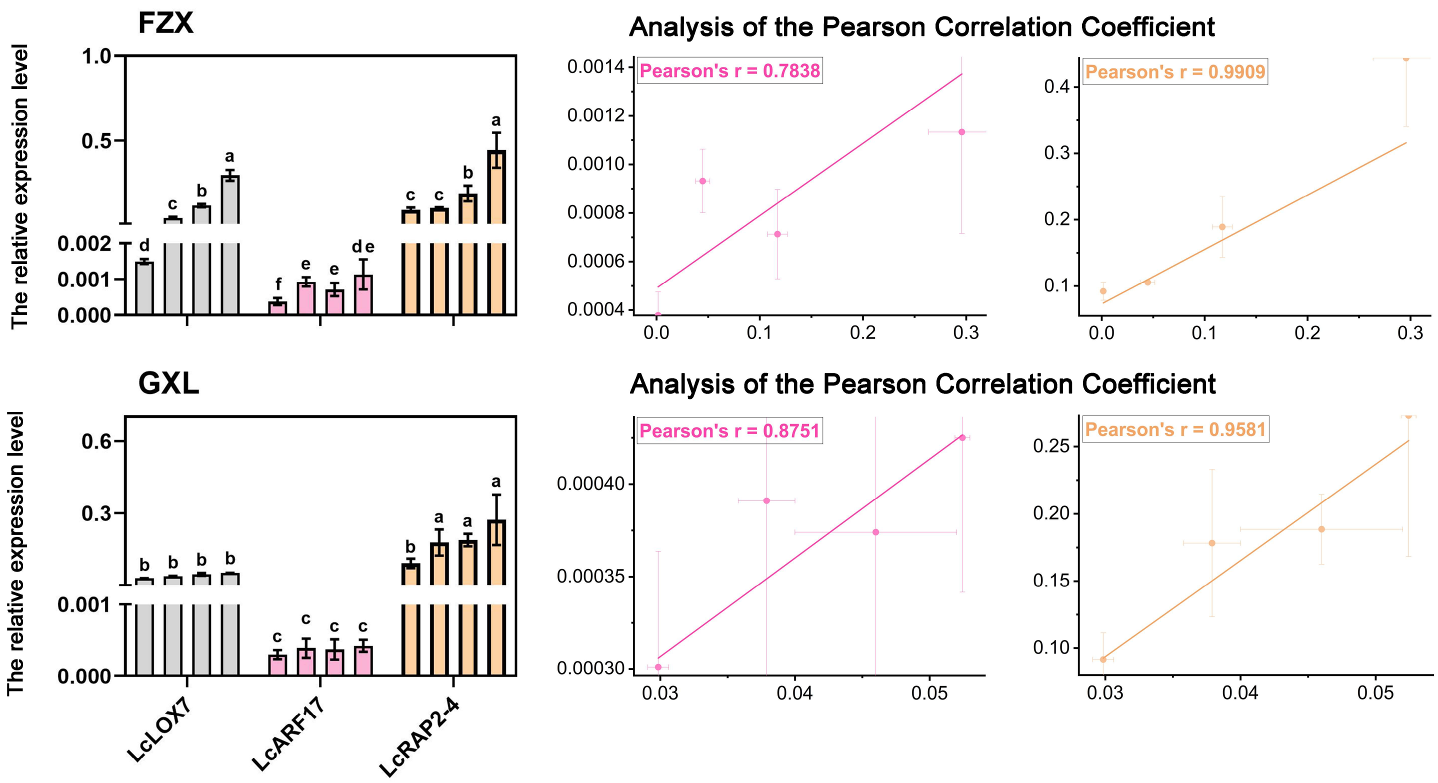

Figure S3. Correlation analysis of RT-qPCR results during fruit maturation in FZX and GXL. The gray bars represent *LcLOX7*, the pink bars represent *LcARF17*, and the orange bars represent *LcRAP2-4.* Statistical significance is indicated by letters. If any letter is the same between groups, it indicates no significant difference; if the letters are different, it indicates a significant difference.

A

B

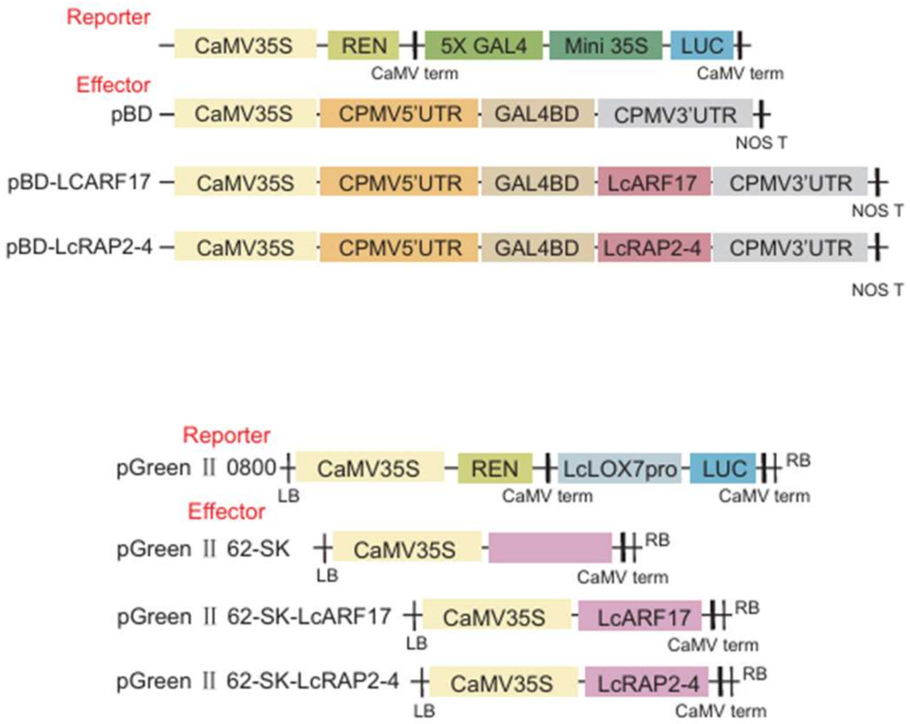

Figure S4. Schematic maps of constructs used in this study. A) Diagram of reporter and effector constructs used in the transcription activity analysis of LcARF17 and LcRAP2-4. B) Diagram of reporter and effector constructs used in the transient assays of LcARF17 and LcRAP2-4, which activate the expression of promoter of *LcLOX7*.

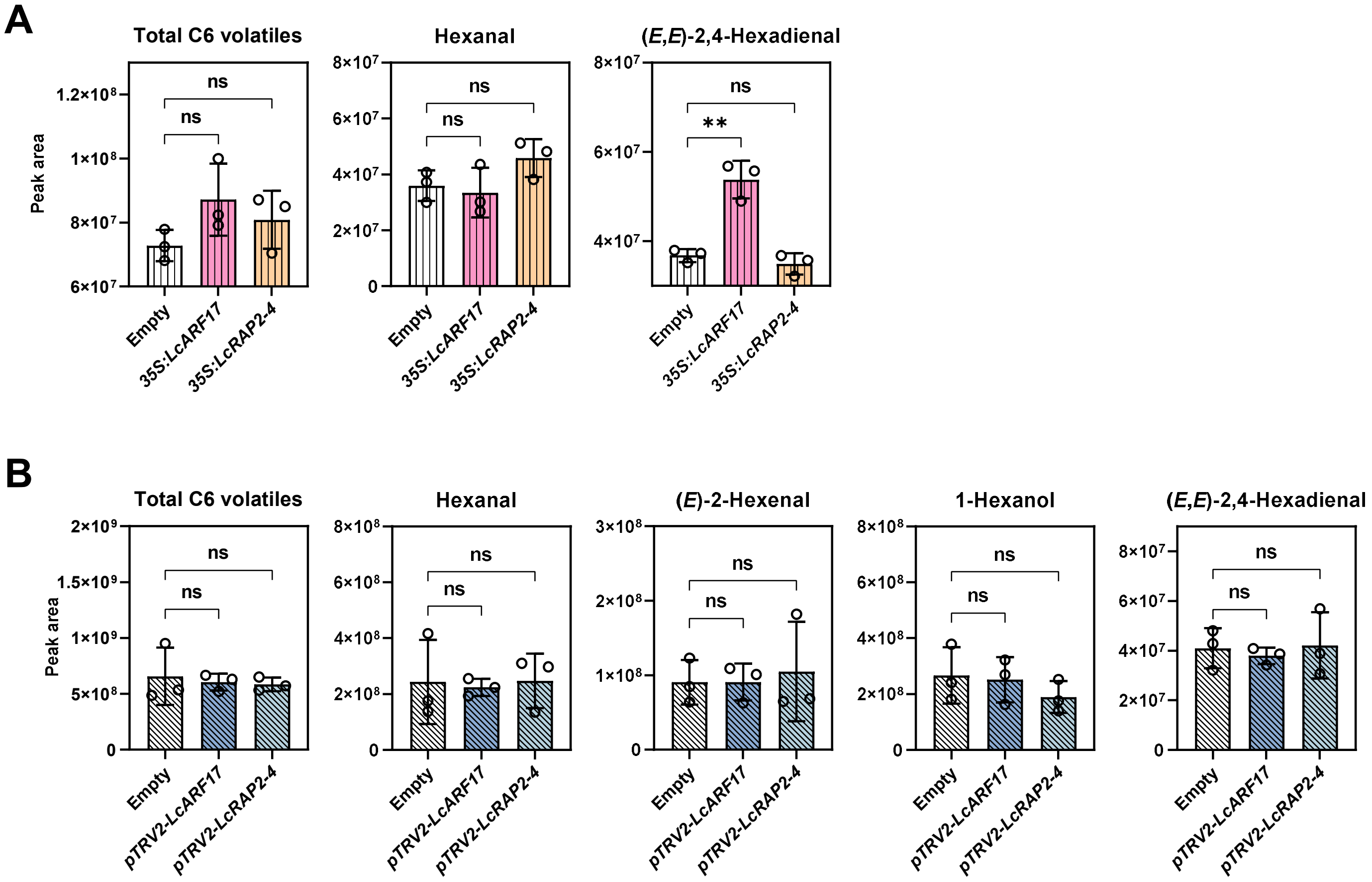

Figure S5. Abundance of C9 volatiles in overexpressed and silenced *LcARF17/LcRAP2-4* litchi callus. A) Abundance of C9 volatiles in *LcARF17*/*LcRAP2-4*-overexpressing litchi callus. Control groups are represented by white-striped bars, *LcARF17*-overexpression by pink-striped bars, and *LcRAP2-4*-overexpression by orange-striped bars. B) Abundance of C9 volatiles in *LcARF17*/*LcRAP2-4*-silenced litchi callus. Control groups are represented by white diagonal-striped bars, *LcARF17*-silenced by blue diagonal-striped bars, and *LcRAP2-4*-silenced by bluish-green diagonal-striped bars. Statistical significance was determined by Student's *t*-test and indicated as follows: * for $p < 0.05$, ** for $p < 0.01$, and ns for not significant.

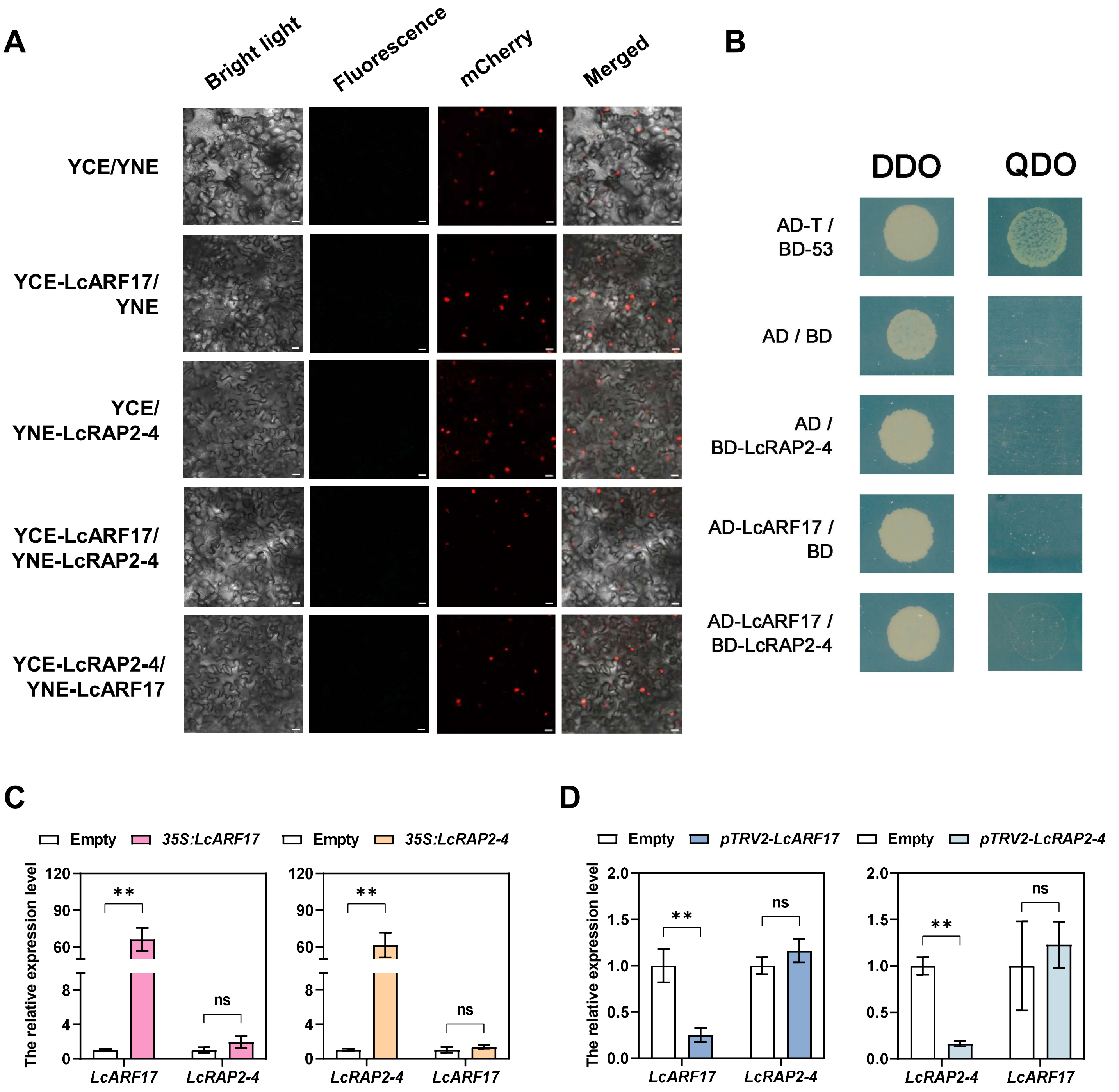

Figure S6. Verification of the interaction and regulatory independence of LcARF17 and LcRAP2-4. A) Bimolecular Fluorescence Complementation verification experiment. The nuclear localization marker (At1g22590) were detected in the mCherry channel. GFP Fluorescence (indicating YFP reconstitution upon interaction) was detected in the green channel. A bright-field image was also captured. Merged images show the overlay of mCherry, Fluorescence, and Bright light channels. Scale bars = 20 µm. B) Yeast two-hybrid verification experiment. DDO represents the SD-trp-leu deficient medium. QDO stands for SD-trp-leu-his-ade deficient medium. Lack of growth on QDO confirms no direct interaction between the two TFs. C) Expression levels of gene-overexpressing litchi callus. Control groups are represented by white bars, *LcARF17*-overexpression by pink bars, and *LcRAP2-4*-overexpression by orange bars. D) Expression levels of gene-silenced litchi callus. Control groups are represented by white bars, *LcARF17*-silenced by blue bars, and *LcRAP2-4*-silenced by bluish-green bars. Statistical significance was determined by Student's *t*-test and indicated as follows: * for $p < 0.05$, ** for $p < 0.01$, and ns for not significant.
